# Suppression of *Schistosoma japonicum* Acetylcholinesterase Affects Parasite Growth and Development

**DOI:** 10.3390/ijms19082426

**Published:** 2018-08-16

**Authors:** Hong You, Chang Liu, Xiaofeng Du, Sujeevi Nawaratna, Vanessa Rivera, Marina Harvie, Malcolm Jones, Donald P. McManus

**Affiliations:** 1 Molecular Parasitology Laboratory, QIMR Berghofer Medical Research Institute, Brisbane 4006, Australia; cwliuchang@gmail.com (C.L.); Xiaofeng.Du@qimrberghofer.edu.au (X.D.); Sujeevi.Nawaratna@qimrberghofer.edu.au (S.N.); Vanessa.Rivera@qimrberghofer.edu.au (V.R.); mcgharvie@gmail.com (M.H.); 2 Parasitology Laboratory, School of Animal Medicine, Northeast Agricultural University, HarBin 150030, China; 3 School of Veterinary Science, The University of Queensland, Gatton 4343, Australia; m.jones@uq.edu.au

**Keywords:** *Schistosoma japonicum*, zoonotic schistosomiasis japonica, acetylcholinesterase, RNA interference, vaccine target

## Abstract

To further investigate the importance of *Schistosoma japonicum* acetylcholinesterase (SjAChE) in cholinergic signaling for parasite growth and development, we used RNA interference (RNAi) to knock-down its expression in adults and eggs in vitro. This resulted in its reduced transcription but also expression of other important genes involved both in cholinergic signaling and glucose uptake were impacted substantially. Significant decreases in AChE protein expression, AChE enzymatic activity, and glucose uptake were observed in the *SjAChE*-knockdown parasites compared with luciferase controls. In vaccine/challenge experiments, we found that immunization of mice with recombinant SjAChE (rSjAChE) expressed in *Escherichia coli* elicited reductions in male worm numbers (33%), liver granuloma density (41%), and reduced numbers of mature intestinal eggs (73%) in the vaccinated group compared with the control group. These results indicate AChE plays an important role in the metabolism of male worms, and impacts indirectly on female fecundity leading to increased numbers of immature eggs being released and reduced sizes of liver granulomas. Furthermore, cytokine analysis showed that immunization of mice with rSjAChE elicited a predominantly Th1-type immune response characterized by increased production of IFNγ in splenic CD4^+^ T cells of vaccinated mice. The study confirms the potential of SjAChE as a vaccine/drug candidate against zoonotic schistosomiasis japonica.

## 1. Introduction

Schistosomiasis remains one of the most insidious and serious of the tropical parasitic diseases of clinical and public health significance. Currently, there is no effective vaccine to prevent schistosomiasis [1] and treatment relies heavily on a single drug, praziquantel (PZQ). There is concern that the widespread use of PZQ may lead to a high risk of drug resistance developing in the future in endemic areas. It is noteworthy that components of the neuromuscular system are the targets of a number of currently approved and marketed anthelminthics [2], including levamisole, pyrantel, monepantel [2,3], and metrifonate [4]. This is based on the critical role the neuromusculature plays in the interaction of helminth parasites with the host and external environments. However, metrifonate, which targets schistosome acetylcholinesterase (AChE) and effectively kills *Schistosoma haematobium* [5], was withdrawn from the market [6] because of an unacceptable level of toxicity to the host and the variable efficacy against other schistosome species [7].

It is known that cholinergic mechanisms in schistosomes [8] are associated with neuromuscular function [9,10], and are highly involved in muscle activity and other essential activities, including host attachment, feeding, and mating [11]. In the flatworm cholinergic system, AChE plays an important role in regulating the interaction between acetylcholine (ACh) and the parasite nicotinic acetylcholine receptors (nAChRs) [12] by hydrolyzing ACh to choline and acetate, allowing ions to pass down electrochemical gradients into or out of cells [13].

To date, AChE has been characterized from *S. mansoni*, *S. haematobium*, *S. bovis*, and *S. japonicum* [12,14,15], which show the enzyme is expressed in high levels on the schistosome tegumental membrane [15,16] and in the musculature, both in blood dwelling adults and the invading larvae, the schistosomula. These characteristics raise the possibility of a role for AChE other than in termination of synaptic transmission and also as a potential immunological target. A recent schistosome protein microarray study showed a predicted *S. japonicum* AChE precursor (AY810792) was significantly targeted by protective IgG1 immune responses in *S. haematobium*-exposed individuals that had acquired drug-induced resistance to schistosomiasis after PZQ treatment [17], thereby supporting consideration of AChE as a suitable antischistosomiasis vaccine candidate. Importantly, the absence of cross-reactivity with human AChE further supports schistosome AChE as an encouraging target for immunological attack [10]. In vitro studies have shown that polyclonal anti-AChE antibodies are cytotoxic and cause complement-dependent killing of 85% of schistosomula [10,18].

To further investigate the biological functions of *S. japonicum* AChE (SjAChE), we employed RNA interference (RNAi) to explore its functional roles in adults and eggs. To determine whether rSjAChE could generate repression in worm growth and fecundity, we carried out vaccine trials in murine vaccine/challenge experiments with *S. japonicum* and measured the immune response generated by rSjAChE to further establish its potential as a vaccine target.

## 2. Results

### 2.1. RNAi-Induced Knockdown of SjAChE

To determine whether knock-down of *SjAChE* resulted in gene silencing in *S. japonicum* via RNAi, purified liver eggs and fragmented and intact adults were electroporated with *SjAChE* dsRNAs and irrelevant luciferase dsRNA (ds*LUC*). The different phenotype changes in the parasites following knock-down of *SjAChE* are now described.

#### 2.1.1. RNAi-Induced Knockdown of *SjAChE* Regulated Transcription of Nicotinic Acetylcholine Receptors and Genes Involved in Glucose Uptake

**Figure 1:**
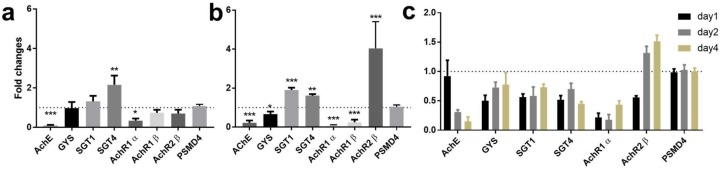
Modulation of transcript levels of nicotinic acetylcholine receptors and genes involved in glucose uptake in (**a**) fragmented and (**b**) intact adult *S. japonicum* and (**c**) eggs after treatment with *SjAChE* dsRNAs. mRNA levels of these genes, relative to the house-keeping gene *PSMD4*, were analyzed by qRT-PCR. Data are representative of the mean ± SEM of two separate experiments. *p* values were calculated using one-way ANOVA to compare the gene fold changes between each RNAi group and the luciferase RNAi control group. * = *p* value ≤ 0.05, ** = *p* value ≤ 0.001, *** = *p* value ≤ 0.0001; *p* value comparing the fold changes between the targeted gene and PSMD4.

Real-time PCR was performed targeting *SjAChE* and other *S. japonicum* genes implicated in cholinergic signaling (nicotinic acetylcholine receptors; *AChR1α*, *AChR1β*, and *AChR2β*) and genes involved in glucose import (*SGT1* and *SGT4*) and glycogen synthesis (*GYS*). Figure 1 shows the transcript levels of these six genes on day 2 after electroporation in fragmented (Figure 1a) and on day 4 after electroporation in intact (Figure 1b) adult *S. japonicum*. The transcript levels of these genes (except *AChR1β*, which was expressed at a very low level in eggs) in dsRNA-treated eggs on day 1, 2, and 4 post-electroporation are shown in Figure 1c.

Compared with controls, we found adult worms treated with dsRNA exhibited a 95% decrease in the level of expression of *SjAChE* (*p* = 0.0006, Figure 1a) on day 2 in fragmented adults and a 80% (*p* = 0.0007, Figure 1b) reduction in *SjAChE* transcription on day 4 in intact adult worms. When fragmented adult *S. japonicum* were treated with *SjAChE*-dsRNA for 2 days, a decrease in gene expression of *AChR1α* (70%, *p* = 0.016) and an increase in *SGT4* expression (2-fold, *p* = 0.0015) were evident. Intact adult worms treated with *SjAChE*-dsRNAs exhibited a pronounced reduction in expression of *GYS* (36%, *p* = 0.028), *AChR1α* (96%, *p* ˂ 0.0001), and *AChR1β* (76%, *p* = 0.0003) but increased expression of *SGT1* (1.8-fold, *p* ˂ 0.0001) and *SGT4* (1.6-fold, *p* = 0.004), *AChR2β* (3.8-fold, *p* = 0.048) on day 4.

Eggs treated with SjAChE-dsRNAs for 1, 2, and 4 days exhibited a consistent reduction in expression of *SGT1* and *SGT4* and *AChR1α* over the time course, whereas *GYS* expression was decreased on days 1 and 2, but returned to the original expression level on day 4 (Figure 1c). *AchR2β* expression was unaffected on day 1 but increased on days 2 and 4; *SjAChE* expression was reduced by 69% (*p* = 0.0006) on day 2 and by 85% (*p* = 0.0001) on day 4 (Figure 1c).

The wild type egg and worm controls had a similar level of transcription for these genes over the 4-day time course as was also evident with the dsLUC control.

#### 2.1.2. Reduction in SjAChE Protein Expression in Parasites Treated with *SjAChE* dsRNA

**Figure 2:**
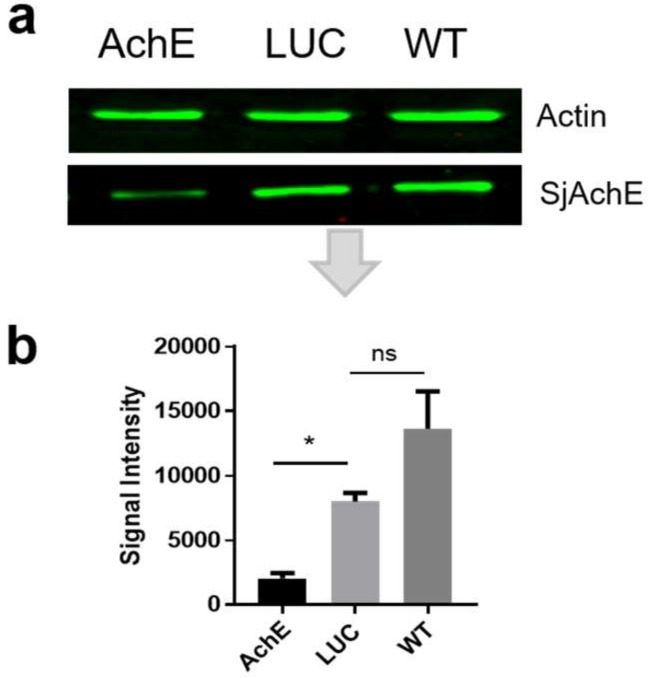
Effect of knockdown of *SjAChE* in intact adult *S. japonicum* worms treated with dsRNA for 4 days determined by Western blotting. (**a**) Anti-actin antibody (upper) and anti-SjAChE antibody (lower) were used to probe a protein extract of intact adult *S. japonicum* 4 days after treatment with *SjAChE* dsRNA (Lane 1); ds*LUC* (Lane 2); and without dsRNA (Lane 3); (**b**) signal intensities of bands recognised by anti-SjAChE antibody in panel a (bottom) were measured using the Odyssey Classic Infrared Imager with a scan intensity setting of 5 and a sensitivity of 5. Error bars represent the standard error of the mean (SEM). This experiment was performed twice. *LUC*, luciferase control group; WT, wild type unsuppressed control worms cultured in the same media; ns, no significant difference. * = *p* value ≤ 0.05.

To determine whether the knockdown of the *SjAChE* dsRNAs was mirrored at the protein level, we performed Western blot analysis using extracts of intact adult worms (SWAP) obtained four days post-treatment with dsRNA. Anti-SjAChE antisera were generated [15] and an anti-actin antibody was used as the primary antibody. Markedly decreased levels of SjAChE protein expression (75%, *p* = 0.0015) were evident in intact adult worms treated with *SjAChE* dsRNA compared with control worms treated with ds*LUC* (Figure 2b), whereas the same level of actin was evident in all adult groups (Figure 2a). There was no significant difference observed between ds*LUC* treated worms and WT (wild type) worms.

#### 2.1.3. Effect of SjAChE Gene Suppression on AChE Activity of Adults and Eggs Treated with SjAChE dsRNA

**Figure 3:**
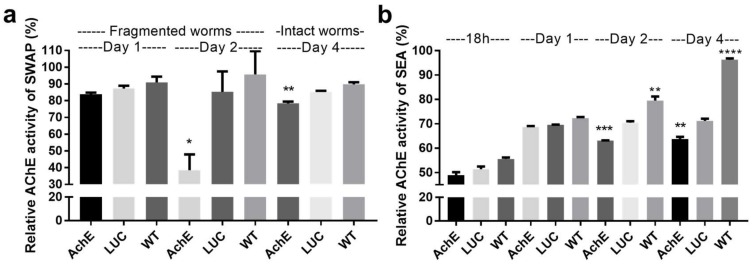
AChE activity of adult *S. japonicum* and eggs after treatment with *SjAChE* dsRNAs at different time points. (**a**) AChE activity of fragmented adult *S. japonicum* on days 1 and 2 and AChE activity of intact adults after treatment with *SjAChE* dsRNAs on days 4; (**b**) AChE activity of *S. japonicum* eggs after treatment with *SjAChE* dsRNAs at 18 h and 1, 2, and 4 days. Data are representative of the mean ± SEM of three separate experiments. *p* values were determined using *t*-tests to compare the differences between the luciferase RNAi control group and each RNAi group or each WT control group. * = *p* value ≤ 0.05, ** = *p* value ≤ 0.001, *** = *p* value ≤ 0.0001.

The AChE activities of *SjAChE*-suppressed adult worms and eggs were compared with ds*LUC* knockdown and unsuppressed worms (wild type, WT) controls at different time points. A significant decrease in AChE activity (54%, *p* = 0.01) was observed in *SjAChE*-suppressed fragmented worms until day 2 after dsRNA treatment compared with the ds*LUC* control group (Figure 3a). In intact adult *S. japonicum*, there was only an 8% reduction in AChE activity (*p* = 0.003) induced on day 4 after dsRNA treatment (Figure 3a). There was no significant change in AChE activity of adults detected between ds*LUC* and WT groups (Figure 3a). The AChE activity of *SjAChE*-suppressed eggs was compared with ds*LUC* knockdown and unsuppressed egg (WT) controls after 18 h, 1 day, 2 days, and 4 days post-electroporation. There was a significant decline in AChE activity (10.5%, *p* = 0.0002) detectable on day 2 and this reduced level of activity was maintained until day 4 (10.4%, *p* = 0.0034) after dsRNA treatment (Figure 3b), compared with the enzyme activity in ds*LUC*-treated eggs. However, AChE activity was found to be increased in WT eggs when the culture time was extended to day 2 and 4 compared with the ds*LUC* group, indicating the electroporation alone may have affected egg secretion of AChE (Figure 3b).

#### 2.1.4. Effect of *SjAChE* Gene Suppression on Glucose Uptake by Adults

**Figure 4:**
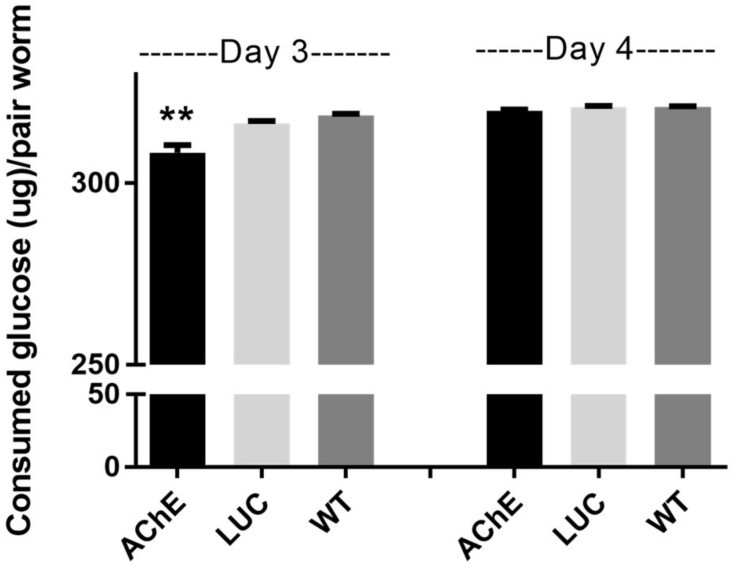
Glucose consumed by intact adult worm pairs of *S. japonicum* after treatment with *SjAChE* dsRNAs on days 3 and 4. Data are representative of the mean ± SEM of three separate experiments. *p* values were calculated using *t*-tests to compare the difference between *SjAChE* knock-down group and the luciferase RNAi control group. ** = *p* value ≤ 0.001.

Glucose uptake in *SjAChE*-suppressed intact adult worm pairs was compared with ds*LUC* knockdown and unsuppressed worm controls. Glucose consumed by each worm pair decreased significantly by 2.5% (*p* = 0.025) in worms treated with *SjAChE* dsRNAs after 3 days incubation compared with the ds*LUC* treated and untreated (WT) control groups (Figure 4). However, at 4 days post-treatment, there was no longer any difference in the glucose consumed by the *SjAChE* dsRNA-treated group compared with the ds*LUC* knockdown control group (Figure 4). There was no difference in consumed glucose by either the untreated worms or by worms treated with ds*LUC* on days 3 and 4 (Figure 4).

### 2.2. Immunolocalization of SjAChE in Eggs Trapped in Liver 

**Figure 5:**
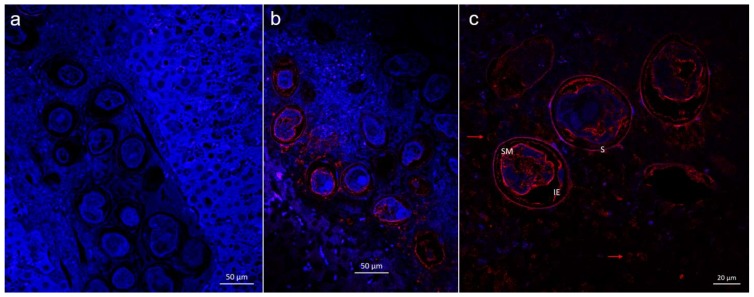
Immunofluorescence of SjAChE in eggs trapped in infected mouse liver probed with rabbit anti-SjAChE antibody. Immunohistological detection of SjAChE in eggs and granulomas in a *S. japonicum*-infected mouse liver. Sections of livers from mice infected with *S. japonicum* were incubated with: (**a**) naïve control rabbit serum; (**b**,**c**) rabbit anti-rSjAChE antiserum; and subsequently with Alexa fluor^®^ 647 donkey anti-rabbit IgG (red fluorescence). DAPI stained nuclei are blue. Section of schistosome eggs in a developing granuloma shows SjAChE staining (red) in eggs and around some circum-oval granuloma cells at 20× magnification (**b**) and 40× magnification (**c**). SM-Surface of miracidium; IE-Inner envelope; S-Shell; red arrow—circum-oval cells in the granulomas.

Immunofluorescence of eggs trapped in infected mouse livers showed that native SjAChE was localized between the egg shell and miracidium with signal localized within the extra embryonic envelopes, the outer envelope and the cellular inner envelope of the egg (von Lichtenberg’s layer) [19,20]. It was also present both on the ciliated surface of miracidia and internally in cell masses (Figure 5). Furthermore, SjAChE staining was detected in the vicinity of the eggs around and even inside some circum-oval cells in the inner most cell layer of advanced egg granulomas (Figure 5).

### 2.3. Vaccine Efficacy of the SjAChE Protein Induced in Inbred CBA Mice

#### 2.3.1. Worm and Egg Reduction in rSjAChE Vaccinated Mice after Cercarial Challenge

Parasitological data for the rSjAChE-vaccinated and unvaccinated (control) mice challenged with 34 ± 1 *S. japonicum* cercariae are presented in Table 1. The vaccine elicited 32% (*p* = 0.03) reduction in male worm number, 41% (*p* = 0.009) decrease in liver granuloma density, and 73% (*p* = 0.0006) reduction in mature (developmental stage V) intestinal eggs (Table 1).

#### 2.3.2. Antibody Responses in Vaccinated Mice

Specific antibody (IgG, IgG1, IgG2a, IgG2b, IgG2c, and IgG3) titers in the sera of the rSjAChE vaccinated mice prior to cercarial challenge and prior to perfusion are shown in Table 1. The rSjAChE vaccinated mice generated high levels of specific anti-rSjAChE IgG, IgG1, IgG2a, and IgG2b with comparatively lower IgG2c and IgG3 titers. The levels of specific IgG1 and IgG2a and the ratio of IgG1/IgG2a at 4, 6 (prior to challenge), 8, 10, and 12 (prior to perfusion) weeks post-primary immunization are shown in Table 2. Both IgG1 and IgG2a responses were increased from 4 to 6 weeks post the primary immunization. The level of IgG1 dropped dramatically at weeks 6 to 8, stabilized at this level at weeks 8–10, and then decreased in weeks 10–12. However, the IgG2 titer remained at a peak level in weeks 6–8 weeks and then declined in weeks 10 to 12 (Table 2). Furthermore, the IgG1/IgG2a ratio was reduced from week 4 to 12 after the primary rSjAChE vaccination leading to the possibility that a Th1 type of immune response was induced by the vaccine.

#### 2.3.3. Cytokine Analysis of Vaccinated Mice

Splenocytes isolated from the rSjAChE-vaccinated and control mice were stimulated with rSjAChE, SWAP, and soluble egg antigen (SEA) in vitro and this was followed by intracellular cytokine staining and analysis by multi-color flow cytometry. The response of splenic T cells to the ex vivo antigen sources was estimated as a proportion of cytokine (IFNγ and IL-4) producing cells.

**Figure 6:**
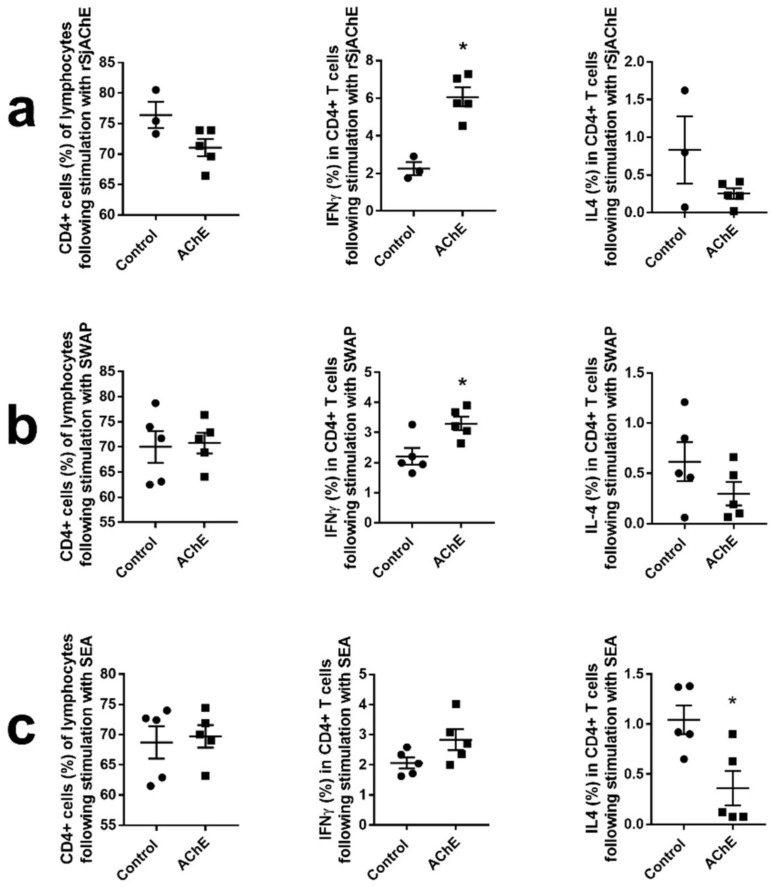
Cytokine profiles of splenocytes recovered from SjAChE-vaccinated and control mice at 6 weeks post- challenge. CD4^+^ T cells as a percentage of total lymphocytes and production of IFNγ and IL-4 in splenic CD4^+^ T cells isolated from vaccinated mice compared with controls following stimulation with (**a**) rSjAChE protein; (**b**) SWAP and (**c**) SEA for 3 days. * = *p* value ≤ 0.05.

The CD4^+^ T cells as a percentage of lymphocytes isolated from rSjAChE vaccinated/unvaccinated mice, following stimulation including rSjAChE, SWAP, and SEA, were determined and are shown in Figure 6 (right panel). We found production of IFNγ in splenic CD4^+^ T cells isolated from vaccinated mice was increased following stimulation with rSjAChE (*p* = 0.0018, Figure 6a) and SWAP *(p* = 0.0159, Figure 6b) compared with cells from control mice. It is noteworthy that there was a decreased production of IL-4 in splenic CD4^+^ T cells *(p* = 0.015, Figure 6c) isolated from vaccinated mice following stimulation with SEA compared with cells from control mice. However, there was no significant difference in the production of IFNγ or IL-4 in splenic CD8^+^ T cells isolated from rSjAChE-vaccinated and unvaccinated mice, when stimulated with AChE, SWAP, or SEA.

## 3. Discussion

Genomic studies of *S. mansoni* [21], *S. haematobium* [22], and *S. japonicum* [23] have stimulated interest in the functional characterization of cholinergic chloride channels and in revisiting the unusual inhibitory activity of AChE and the interaction between ACh and nAChRs in schistosomes, with both types of molecules (ACE and nAChRs) targeted by a number of currently approved and marketed anthelminthic drugs. To better understand the potential roles of AChE and its involvement in the cholinergic system and glucose uptake in schistosomes, we have now shown that RNAi knockdown of *SjAChE* can affect the regulation of a number of key genes in parasite cholinergic signaling (including *nAChR1α*, *1β*, and *2β*) and glucose metabolism (including *SGT1*, *SGT4*, and *GYS*). In this study, the transcriptional suppression of *SjAChE* was more pronounced in fragmented worms on day 2 post-treatment with *SjAChE* dsRNA compared with that of intact worms on day 4. The 95% reduction in expression of *SjAChE* in fragmented adults in a short period (2 days) may have led to higher expression of *SGT4*, in order to import more glucose into *S. japonicum* for survival, whereas by this time the expression of *GYS*-glycogen store had not yet been altered. However, on day 4 post-treatment, intact worms subjected to *AChE* suppression showed a pronounced increase in the transcript levels of *SGT1* and *SGT4* with a decline in *GYS*, indicating a critical role for *SjAChE* which may indirectly affect parasite glucose uptake and storage. This outcome supports a previous study of ours showing that knock-down of key genes (such as *S. japonicum* insulin receptors [24]) which are involved in parasite glucose uptake, strongly stimulated *S. japonicum* worms to express more *SGT* to allow the acquisition of more host glucose as an emergency response in the suppressed worms. The transcription of *AChR1α* and *1β* also decreased in intact adults treated with dsRNA for four days but, notably, *AChR2β* was substantially increased. A similar profile was also observed in eggs following dsRNA treatment for four days.

Recently we identified cDNA sequences of *nAChR1α*, *1β*, and *2β* from *S. japonicum*, but undertook no further characterization of these molecules. It has been reported that *S. haematobium* AChR1α is located on the parasite surface and may contribute to the potentiation of the uptake of glucose from host blood in response to circulating concentrations of ACh [25], whereas AChR1β is distributed within the musculature and on discrete cell bodies within the connective parenchyma [25]. The depressed expression of *AChR1α* in both fragmented and intact adult *S. japonicum* with knock-down of *SjAChE*, indicated the close relationship between AChE and AChR1α, both of which are located in/on the membrane surface of schistosomes and are associated with its transporting function in nutrient transport. Unexpectedly, the transcription of *AChR2β* was highly increased both in adults and eggs when *AChE* was depressed. However, details of cholinergic signaling transduction and the interaction of these molecules remain unknown in schistosomes.

Previous study showed that AChE also plays an important role in helping import glucose from host blood into schistosomes [26] and the influence of ACh (which is present in host blood) on glucose uptake can be reduced through inhibition of either the tegumental AChE [27] or tegumental nAChR [12] of adult worms. In this study, when *AChE* was depressed by dsRNA treatment in intact adults in vitro, the consumed glucose of paired worms showed a small (2.5%), but significant decrease on day 3 post dsRNA treatment. However, this effect of transcriptional suppression of *AChE* on glucose uptake in *S. japonicum* adults disappeared by day 4 post-treatment, indicating the indirect role of *AChE* in regulating parasite glucose uptake. It should be noted, however, that AChE activity was still maintained at a comparatively low level in suppressed worms, suggesting either that the parasites are able to compensate, either directly or indirectly, for loss of an essential metabolic pathway. Schistosomes are complex multicellular and multi-organ organisms and likely use multiple mechanisms to survive in the mammalian host. It is possible that blocking or inhibiting one gene or gene product may stimulate worms to compensate by switching to a subpathway or another alternative but related pathway to ensure their ability to acquire and metabolize glucose from the host [28].

Disruption of *AChE* in schistosomes would likely result in reduced AChE activity levels both in eggs and adults; with the latter this would in turn logically result in depressing metabolism of adult worms by inhibiting neuromuscular activity [11]. This was also reflected in the reduction of male worm numbers in mice vaccinated with rSjAChE shown here. The RNAi experiments showed a significant decrease (54%) in AChE activity in *SjAChE*-suppressed, fragmented adults on day 2, but only an 8% reduction in AChE activity in intact worms on day 4 after dsRNA treatment (Figure 3a). This indicates again that, more effective knock-down can be performed with fragmented parasites, whereas intact worms showed a better capacity to maintain multiple mechanisms for their survival in vitro by switching to an alternative cellular pathway when *SjAChE* was suppressed. In the in vitro RNAi experiments, we observed a 75% of reduction in AChE protein expression (Figure 2), but only an 8% decrease in AChE activity (Figure 3) in the suppressed intact worms on day 4. This could be interpreted as reflecting the impact of the environmental conditions prevailing in vivo on parasite growth which are likely to be far more complicated than the in vitro culture system we used in this study. In the host, the AChE activity of worms might be stimulated or regulated by other components or factors, such as the ACh concentration in the host blood, and/or interactions between Ach and AChRs, or a synergistic effect with other enzymes, yet to be characterized in schistosomes. However, reduced AChE activity of *SjAChE*-suppressed eggs was detectable on day 2 after dsRNA treatment and the same 10% reduction was maintained until day 4 (Figure 3b) compared with ds*LUC*-treated eggs, suggesting stability of *AChE* knock-down efficacy in this inactive stage.

The immunohistological staining showed the SjAChE protein was present in thin cellular epithelia of the egg, the extra-embryonic inner envelope or von Lichtenberg’s envelope, and the ciliated epithelium of the miracidium. Ashton and colleagues [29] postulated that a role for the inner envelope of the egg was to synthesize proteins for transport and storage in the acellular outer envelope (Reynold’s layer) prior to secretion from the egg. Notably, SjAChE staining was observed in cells of the innermost cellular layers of advanced schistosome egg granulomas (Figure 4b) implying that SjAChE might be secreted by the eggs and is taken up by some of the surrounding cells. This view is substantiated by a previous study showing there is no immunological cross-reactivity between AChE from schistosomes and humans [10]. The decreased production of IL-4 in splenic CD4^+^ T cells isolated from rSjAChE-vaccinated mice following stimulation with SEA compared with cells from unvaccinated controls, indicates antibody raised against rSjAChE or the secreted SjAChE around eggs may have inhibited the host IL-4 response.

For the first time, we moved to confirm the important role of SjAChE in the growth and development of adult *S. japonicum* by using a vaccine/challenge strategy we have used previously [30]. Immunization of mice with rSjAChE-QuilA elicited reductions in male worm numbers (33%), liver granuloma density (41%), and mature intestinal eggs (73%) compared with the adjuvant-only group (Figure 5). Given a previous in vitro study showing that polyclonal anti-AChE antibodies cause complement-dependent killing of schistosomula [10], the reduction in male worm burden observed in the rSjAChE vaccinated/challenged mice may be a consequence of immature males being killed at an early stage of infection. AChE is located on the surface and in the musculature of adult schistosomes [15,16] and functions in the motor activity of schistosomes [8]. When AChE activity was inhibited, the higher levels of ACh in host blood caused excessive stimulation of nAChR leading to its desensitisation and closing of ion channels [12] which is critical for parasite muscle function and nerve action. Male schistosomes are far more muscular than females [31] and AChE is predominantly concentrated on the dorsal surface of males [32]; there is only low representation of AChE on the head, tail, ventral surface, and gynaecophoric canal of male worms and the entire female tegument [33]. Immunization of mice with rSjAChE did not induce any reduction in female worm number, but it did depress survival of male *S. japonicum* and impacted pairing and mating, thereby indirectly affecting the fecundity of females and also led to an increase in immature eggs being released and the development of fewer liver granulomas. These results strongly support previous research showing the role of SjAChE in the cholinergic system [8], in neuromuscular function [9,10], and other essential activities in schistosomes, including host attachment, feeding, and mating [11]. Given the indirect role of AChE in helping to import glucose from host blood into schistosomes, these results also indicate combining SjAChE with other vaccine candidate might be a potential strategy to enhance vaccine efficacy.

To determine T cell cytokine production induced by immunization of mice with rSjAChE, T cell populations and their production of IFNγ and IL-4 were assessed using multicolor flow cytometry. At 6 weeks post-challenge, increased production of IFNγ in splenic CD4^+^ T cells isolated from vaccinated mice was observed compared with unvaccinated control mice, following stimulation with either SjAChE or SWAP, indicating high levels of antigen-specific IFN-γ (Th1) were associated with protection against *S. japonicum* infection in mice, in concordance with data from other studies [34,35,36]. The increased production of IFNγ in splenic CD4^+^ T cells observed in the vaccinated mice may inhibit the acceleration of the Th2 response induced by new-laid eggs wrapped in host tissue at 12 weeks after the first immunization (6 weeks post-challenge). This was also reflected in decreased production of IL-4 in splenic CD4^+^ T cells isolated from the vaccinated mice following stimulation with SEA compared with controls (Figure 6c).

Previous studies have shown that the levels of IgG1 and IgG2 antibody subclasses can be used as serological markers to indicate the induction of Th1 (IgG2) and Th2 (IgG1) responses [37]. IgG1/IgG2a ratios of <1.0 or a decreased ratio indicate a predominantly Th1 type of response. Th1 cytokines may be beneficial in preventing schistosomiasis, targeting worms during the early stages of infection, while Th2 cytokines, which are induced by egg antigens following egg deposition in tissues during the late immune response, suppress the Th1 response [38]. Our results showed that protective immunity in mice was associated with high titers of specific anti-SjAChE IgG1 and IgG2a antibodies and a reduced IgG1/IgG2a ratio at eight weeks after the first immunization (Table 2). This finding further confirms the generation of a Th1 type of immune response is induced by rSjAChE vaccination, which matches well with the T cell cytokine analysis discussed earlier.

## 4. Materials and Methods

### 4.1. Ethics Statement

The conduct and procedures involving animal experimentation were approved by the Animal Ethics Committee of QIMR Berghofer Medical Research Institute (project number 288 and ethics ID A0108-054, approval date 31 October 2017). This study was performed in accordance with the recommendations in the Guide for the Care and Use of Laboratory Animals of the USA National Institutes of Health.

### 4.2. Parasites

*Oncomelania hupensis hupensis*, naturally infected with *S. japonicum*, were obtained from an endemic area in Anhui Province, China, and transported to the Brisbane laboratory in Australia. Cercariae were shed from the infected snails and collected as described [39].

### 4.3. Treatment of Parasites with Double Stranded RNA (dsRNA)

Characterization of *SjAChE* gene function was carried out using dsRNA interference as we have previously reported [40]. Swiss mice infected with 35 *S. japonicum* cercariae were euthanized humanely 6 weeks post-infection and adult worms obtained by portal perfusion. Adult *S. japonicum* worms were incubated in Dulbecco’s modified Eagle’s medium (DMEM) (Invitrogen, Carlsbad, CA, USA), supplemented with 10% (*v*/*v*) heat-inactivated foetal calf serum, 100 IU/mL penicillin, and 100 mg/mL streptomycin (complete culture medium) at 37 °C in an atmosphere of 5% CO_2_ in air overnight. dsRNAs targeting on *SjAChE* [15] (GenBank Accession No. KX268651) was synthesized from *S. japonicum* cDNA using gene-targeted primers containing the following T7 promoter sequences: F: 5′-TAATACGACTCACTATAGGGAATTATCGTTTAGGTTCTTTTGGTT-3′; R: 5′-TAATACGACTCACTATAGGGAGGTAGATCAGTCATATTCTTCATCCA-3′. dsRNA was synthesized and purified using a Megascript RNAi kit (Ambion, Foster City, CA, USA). Luciferase dsRNA (ds*LUC*) was used as a negative control, as reported in other RNAi studies with schistosomes [41,42]. In RNAi experiments we used intact but also diced adult worms for dsRNA treatment, as a recent study showed that dicing adult schistosomes into several fragments for RNAi resulted in increased gene reporter activity [43]. Accordingly, freshly perfused adult *S. japonicum* were cultured overnight in complete culture medium. Of those worms, 50% were left intact, while the other 50% were washed and diced into three fragments using a sterile blade. Then, intact and fragmented worms were placed separately in 0.4 cm cuvettes (BIO-RAD and transferred medium was replaced with 50 µL cold Opti-MEM medium containing 25 µg dsRNA). The parasites were subjected to square wave electroporation (125 V, 20 ms, one pulse [40]). After electroporation, worms were transferred to prewarmed complete culture medium, and incubated at 37 °C under 5% CO_2_ in air. Fragmented adults were harvested on day 2 after electroporation, while they still had good motor activity, and intact worms were collected on day 4.

Eggs were isolated from livers of infected mice as described [44], and the purified eggs were incubated overnight in complete DMEM medium before dsRNA treatment. The eggs were washed 3 times with cold Opti-MEM media and then placed into 0.4 cm cuvettes (BIO-RAD, Hercules, CA, USA) (5000 eggs/50 μL reaction) for electroporation, as described above. After electroporation, the eggs were transferred into DMEM complete culture medium and incubated at 37 °C under 5% CO_2_ in air. Eggs were harvested at 18 h, 24 h (day 1), 48 h (day 2), and 96 h (day 4) after electroporation and stored at −80 °C until used for extraction of total RNA and protein.

For each group, 20–25 pairs of adults or 20,000–50,000 eggs in total were treated. Untreated intact or fragmented adult *S. japonicum* and eggs without electroporation were cultured under the same conditions as additional controls (wild type (WT) controls). The RNAi experiments were repeated independently three times.

### 4.4. Glucose Measurement and AChE Activity Assays

Medium (20 μL) in which intact adult worms were cultured was collected on days 3 and 4 after electroporation and the glucose concentration measured using glucose assay kits (Biocore, Gaithersburg, MD, USA), according to the manufacturer’s instructions. Cultured adult worms were collected on day 4 after electroporation for protein extraction of soluble worm antigen preparation (SWAP) [24]. Eggs were collected after 18 h, and on days 1, 2, and 4 post-electroporation and soluble egg antigen (SEA) was prepared as previously described [45]. SEA (at a concentration of 0.45 μg/mL) and SWAP (at a concentration of 7.5 μg/mL) were used for determining AChE activity using the Amplex Red Acetylcholine/Acetylcholinesterase Assay Kit (Invitrogen) according to the manufacturer’s instructions. SWAP samples were also subsequently used for Western blot analysis as described below.

### 4.5. Expression Analysis of SjAChE and Related Genes

Total RNA was extracted from adult worms and eggs harvested from the dsRNA-treated and untreated groups at different time points using Qiagen RNeasy kits (Qiagen, Hilden, Germany). First-strand cDNA was synthesized from total RNA using a Sensiscript Reverse Transcription for First-stand cDNA synthesis Kit (Qiagen) with oligo(dT)15 primers. The cDNA was subsequently used as template in real time PCR analysis to determine the transcriptional levels of several key genes including *SjAChE*, *AchR1α* (nicotinic acetylcholine receptor 1α), *AchR1β*, *AchR2β*, *SGT1* (Glucose transporter protein 1), *SGT4* (Glucose transporter protein 4), and *GYS* (Glycogen synthase). *PSMD4* (26S proteasome non-ATPase regulatory subunit 4) was used as reference gene [46]. Primers were designed using Primer 3 software (http://frodo.wi.mit.edu/) and a primer melting temperature of 55–62 °C. All primer sequences used are shown in Supplementary Table S1. The specificity of each primer sequence was confirmed by BLAST analysis. All qPCR experiments were performed with QuantiNova SYBR Green PCR Kits (Qiagen, Hilden, Germany) under the following conditions: 95 °C for 10 s, 40 cycles of amplification (95 °C for 10 s, 55–62 °C for 40 s), 95 °C for 15 s, 60 °C for 1 min, and 95 °C for 15 s. The results were analyzed using Rotor-Gene 6000 software. Relative expression levels were normalized to *PSMD4* and calculated using the 2^−ΔΔ*C*t^ method [47].

### 4.6. Western-Blot Analysis

RNAi-treated intact adult parasites were harvested four days after electroporation and then lysed with 1% (*v*/*v*) Triton X-100 in Tris buffered saline supplemented with complete protease inhibitor cocktail (Sigma-Aldrich, St Louis, MO, USA) [24]. Rabbit anti-SjAChE serum, prepared as described earlier [15], was used in Western blotting to probe the native SjAChE protein in electrophoresed SWAP. The SWAP samples from dsRNA-treated and untreated groups were separated on 15% (*w*/*v*) SDS-PAGE gels and transferred to Immun-Blot^®^ low fluorescence-PVDF membranes. Overnight blocking was performed with Odyssey buffer containing 2% (*v*/*v*) goat serum at 4 °C. Then, the membranes were probed with the rabbit anti-SjAChE anti-serum (1:100 dilution in Odyssey buffer and 0.1% Tween-20) for 1 h followed by incubation with IRDye-labeled 680LT goat anti-rabbit IgG antibody (Li-COR Biosciences) (1:15,000 diluted in Odyssey buffer with 0.1% Tween-20 and 0.01% SDS) for 1 h on a shaker in a dark chamber. An anti-actin antibody (Sigma-Aldrich) (1:150) [48] was used to assess any protein-loading differences. After a final wash with distilled water, membranes were allowed to dry in the dark and visualized using the Odyssey^®^ CLx Infrared Imaging System [15].

### 4.7. Immunolocalization of SjAChE in Eggs

Paraffin blocks were made from portions of liver excised from infected mice (6 weeks post-cercarial challenge) and tissue sections (4 µM) were cut and placed on charged adhesive microscope slides. Following deparaffinization and rehydration, antigen retrieval was done with Revealt A solution (Biocare Medical, Concord, MA, USA), as previously described [19]. The tissue sections were then blocked with 1% (*w*/*v*) bovine serum albumin and 1% (*w*/*v*) donkey serum in Tris buffered saline (TBS) for 60 min at room temperature in a humidified chamber and incubated with anti-SjAChE antibody (1:200) at 4 °C overnight. After three washes with TBS-T (TBS containing 0.05% (*v*/*v*) Tween 20), the sections were incubated with Alexa fluor^®^ 647 donkey anti-rabbit IgG (1:500) (Invitrogen, Carlsbad, CA, USA) at 37 °C for 1 h. Nuclei in the tissue sections were counterstained with diamidino-2-phenylindole (DAPI) gold (Invitrogen, Carlsbad, CA, USA) and visualized under fluorescence using a Zeiss 780 NLO confocal microscope (Zeiss, Oberkochen, Germany).

### 4.8. Protective Efficacy of the Recombinant SjAChE Vaccine

#### 4.8.1. Recombinant Protein Expression and Purification 

Recombinant SjAChE was expressed in the *Escherichia coli* expression system. A fragment encoding the *SjAChE* (G71-Y537) was amplified by PCR and ligated into the pET28b vector (Invitrogen) by using forward (5′-CGGGATCCGGGAATTTACTGTGGACAACGTG-3′ with *Bam*HI restriction site underlined) and reverse (5′-CGCTCGAGATAACCATGCATTGTACCAGTCC-3′ with *Xho*l restriction site underlined) primers. *E. coli* Rosetta™ (DE3) competent cells (Invitrogen) were transformed with the recombinant plasmids for expression and purification as described [30]. The expressed SjAChE protein was purified from the *E. coli* lysate using Ni-NTA affinity chromatography (GE Healthcare Life Science, Chicago, IL, USA) under denaturing conditions, using 6 M guanidine-HCl and then refolding in buffer (300 mM NaCl, 50 mM NaH_2_PO_4_, 8% *w*/*v* sucrose, PH 4.5). Residual endotoxin was removed from purified proteins as described [30] and assessed by using an Endotoxin (*E. coli*) Standards kit (Lonza, Anaheim, CA, USA). The purified rSjAChE was used in mice vaccine/challenge experiments.

#### 4.8.2. Mice Vaccination

Two groups of female CBA mice (6–8 weeks old, 10 mice/group) were immunized subcutaneously (s.c.) with 25 μg rSjAChE in 0.1 mL of PBS conjugated with 15 μg Quil A adjuvant (InvivoGen, San Diego, CA, USA) and then were boosted s.c. twice at 2 week intervals with the same vaccine regimen. The control mouse group received PBS formulated with Quil A for the primary and two adjuvant boosts by s.c. route. All mice were challenged with 34 ± 1 *S. japonicum* cercariae, counted under a microscope, by the abdominal skin route 2 weeks after the third injection.

#### 4.8.3. ELISA

Serum samples were collected at 0, 2, 4, 6, 8, 10, 12, and 14 weeks after the first immunization to assess IgG, IgG1, IgG2a, IgG2b, IgG2c, and IgG3 antibody responses induced in the rSjAChE-vaccinated mice by ELISA [29]. Briefly, immunoplates (Nalge Nune International, Rochester, NY, USA) were coated with rSjAChE fusion protein (10 μg/mL, 100 μL/well) at 4 °C overnight followed by blocking with blocking buffer (1% BAS in PBST) at 37 °C for 1 h. All serum samples, serially diluted with blocking buffer (100 μL/well), were added to the wells and the plates incubated at 37 °C for 1 h. HRP-conjugated sheep anti-mouse IgG, IgG1, IgG2a, IgG2b, IgG2c, and IgG3 antibodies (Invitrogen, Carlsbad, CA, USA) were then added (1:2000, 100 μL/well) and the plates were incubated for 1 h at 37 °C. Streptavidin-HRP (BD Pharmingen, San Jose, CA, USA) (1:10,000) was then applied to each well (100 μL/well). PBST washes were applied five times after each step, with 2 min between each step. Reactions were developed using TMB substrate (100 μL/well) for 5 min and stopped using 2 M sodium hydroxide (50 μL/well), as previously described [49]. Optical density (OD) values were read at 450 nm using a microplate reader, and all tests were run in duplicate on each test plate. A positive antibody response was defined as an OD value higher than 2.1 times the mean OD values of the serum samples from control mice.

#### 4.8.4. Worm and Egg Counting and Pathology

Worm numbers and egg burdens in livers, intestines, and in feces were determined, as previously described [30,50], in the control and vaccinated mice at six and eight weeks post cercarial challenge. Adult worm numbers were counted and all adult worms from each mouse were fixed and used for length measurement, as previously described [29]. Livers of five mice from each of the vaccinated and control groups were selected randomly for quantitative analysis of the hepatic response to embolized eggs. For each mouse, the left quadrate lobe of each liver was fixed in 4% (*v*/*v*) formalin. Paraffin-embedded sections of these samples were prepared and stained with Haematoxylin and Eosin (H&E). Slides were digitized using an Aperio Slide Scanner (Aperio Technologies, Vista, CA, USA) and the degree of liver pathology was quantified by measurement of the volume density of granulomatous lesions using Aperio ImageScope v11.1.2.760 software (Leica Biosystems Imaging, Buffalo Grove, IL, USA), which was estimated from the area of the granulomas, divided by the total area of the liver tissue in the image. To measure this, four regions of the same magnification and size were taken from each of two liver sections per mouse. Counts for each photograph were averaged for each group. To compare differences in the maturity of intestinal eggs between the SjAChE-vaccinated and control mice, *S. japonicum* eggs were isolated from whole intestinal tissue, as previously described [30,51]. The intestinal egg mixture was fixed in 4% (*v*/*v*) formalin, stained and mounted on microscope slides using Prolong Gold Antifade Reagent incorporating DAPI (Invitrogen) and incubated at 4 °C overnight. The stained eggs were then visualized by fluorescence microscopy (IM1000, Leica, Cambridge, UK) using UV illumination to visualize nuclei. Embryonic development of intestinal eggs obtained from the vaccinated and control mice was compared using a published staging system [52].

#### 4.8.5. Flow Cytometry Analysis

All vaccinated mice were euthanized six weeks post-cercarial challenge. The spleens of each animal (*n* = 5 per group) were collected and splenocytes were isolated as described [53]. Briefly, the spleens were pressed through a 70 µm cell strainer and red blood cells were lysed using RBC lysis buffer (Sigma-Aldrich). Following repeated washing and centrifugation steps, the cells were resuspended in IMDM (Iscoves Modified Dulbecco’s Medium) containing 10% (*v*/*v*) fetal bovine serum, 100 μg/mL Streptomycin, 100 Units/mL Penicillin and 0.05 mM 2-Mercaptoethanol. Cell counts were determined by trypan blue exclusion, and then cells were seeded into 96-well plates (5 × 10^5^ cells/well). The cells were incubated in the presence of SWAP, SEA, or rSjAChE (each, 2 μg/well) for 72 h at 37 °C. Stimulation with ConA (0.1 μg/well) was performed as a positive control and nonstimulated splenocytes were used as a negative control. Brefeldin A (BFA) (2 µg/well) was added to the cell cultures 6 h before harvesting.

The collected cells were then blocked with anti-FcR (2.4G2) and stained with BD Horizon™ fixable viability stain 780 (all from BD Biosciences, North Ryde, Australia) and labeled with anti-mouse CD3-BV421, CD4-Alexa Fluor700, and CD8 BV650 antibodies. Cells were then fixed and permeabilized using BD cytofix/cytoperm buffer according to the manufacturer’s instructions and stained for intracellular expression of cytokines using anti mouse IL-4 PE CF594 and IFNγ-PerCp Cy5.5 (BD Biosciences); corresponding fluorescence minus one controls were also prepared. Samples were collected on a BD Fortessa 4 Laser flow cytometer using FACS DIVA and the results were analyzed using Flowjo 9 software (FLOWJO.LLC, Ashland, OR, USA). GraphPad Prism version 6 was used for all statistical analysis.

### 4.9. Statistical Analysis

All data are presented as the mean ± S.E. Differences between groups were assessed for statistical significance using the *t*-test and One-way ANOVA. A statistically significant difference for a particular comparison was defined as a *p* value ≤ 0.05. GraphPad Prism software (Version 7, GraphPad Software, La Jolla, CA, USA) was used for all statistical analyses. * = *p* value ≤ 0.05, ** = *p* value ≤ 0.001, *** = *p* value ≤ 0.0001.

## 5. Conclusions

We used RNA interference (RNAi) to silence SjAChE in adult worms and eggs in vitro, and undertook vaccine/challenge trials in mice to investigate its role in the growth and development of *S. japonicum*. The results obtained further support the potential of AChE as a future drug or vaccine target against *S. japonicum* infection and also strengthen the view that immunological targeting of schistosome AChEs may be a suitable avenue for developing future treatments and the prevention of schistosomiasis.

## Figures and Tables

**Table 1:** Parasitologic and antibody data for vaccinated and control mice challenged with 34 ± 1 *Schistosoma japonicum* cercariae.

Group	Immunoglobulin		Number Adult Worms Mean ± S.E.	Mean Length of Adult Worms (mm) Mean ± S.E. % Reduction (*p* Value)	Liver Eggs/g Mean ± S.E. % Reduction (*p* Value)	Liver Granuloma Density (%) Mean ± S.E. % Reduction (*p* Value)	Intestinal eggs/g Mean ± S.E. % Reduction (*p* Value)	Maturity of Intestinal Eggs in Stage V (%) Mean ± S.E. % Decrease (*p* Value)					Faecal Eggs/g/F Mean ± S.E. % Reduction (*p* Value)
	Before Challenge	Before Perfusion						Stage I	Stage II	Stage III	Stage IV	Stage V	
Control	IgG, IgG1, IgG2a, IgG2c. and IgG3 1:50	-	(F) 5.9 ± 0.7	(F) 10.4 ± 0.2	41,886 ± 6001	30 ± 3.9	64,483 ± 9998	3.4 ± 1.8	15.6 ± 3	27.7 ± 2	31.6 ± 2	21.6 ± 2	1398 ± 474
			(M) 8.5 ± 0.8	(M) 6.6 ± 0.2									
rSjAChE	IgG 1:819,200 IgG1 1:409,600 IgG2a 1:819,200 IgG2b 1:409,600 IgG2c 1:12,800 IgG3 1:6400	-	(F) 5.3 ± 1.05	(F) 9.8 ± 0.17	38,335 ± 7280	17.8 ± 2 41% ** *p* = 0.009	61,724 ± 12,008	9.8 ± 3.3	25 ± 3.7	34 ± 2.6	25 ± 0.9 ↓21% * p = 0.02	5.8 ± 0.8 ↓73% *** *p* = 0.00006	1039 ± 480 25.6% ns *p* = 0.77
			(M) 5.8±0.9 32% * *p* = 0.03	(M) 6.8 ± 0.19									

Note: F, female worm; M, male worm; n, the number of mice per group that survived the trial and were necropsied; ns, not significant. * = *p* value ≤ 0.05, ** = *p* value ≤ 0.001, *** = *p* value ≤ 0.0001.

**Table 2:** IgG1 and IgG2a immune profile in mice induced by vaccination with recombinant SjAChE and challenge of *S. japonicum.*

Weeks	OD_450_				IgG1/IgG2a Ratio
	IgG1		IgG2a		
	rSjAChE	Control	rSjAChE	Control	
4 (Third-vaccination)	1.237 ± 0.07	0.13 ± 0.0035	0.859 ± 0.024	0.14 ± 0.002	1.441
6 (Pre-Challenge)	1.4 ± 0.016	0.15 ± 0.0005	1.382 ± 0.022	0.15 ± 0.0005	1.013
8	0.98 ± 0.025	0.14 ± 0.0006	1.303 ± 0.003	0.13 ± 0.002	0.751
10	0.99 ± 0.003	0.14 ± 0.0007	1.21 ± 0.029	0.13 ± 0.0006	0.819
12 (Pre-Perfusion)	0.46 ± 0.007	0.16 ± 0.0004	0.827 ± 0.06	0.13 ± 0.0007	0.56

## Supplementary Materials

The following are available online at http://www.mdpi.com/1422-0067/19/8/2426/s1.
